# Life-saving staged management of a secondary aortoesophageal fistula after arch replacement: a case report

**DOI:** 10.1186/s44215-026-00267-0

**Published:** 2026-06-17

**Authors:** Yuki Kamikawa, Yoichiro Machida, Hiromi Fujii, Hiroaki Hata, Shizuyuki Dohi

**Affiliations:** 1 Department of Cardiovascular Surgery, Toda Chuo General Hospital, Saitama, Japan; 2 Department of Cardiovascular Surgery, Juntendo University, Tokyo, Japan; 3 Department of Cardiovascular Surgery, Juntendo University Nerima Hospital, Tokyo, Japan

**Keywords:** Aortoesophageal fistula, Thoracic endovascular aortic repair, Total arch replacement, Oesophagectomy, Omental flap, Reconstruction, Case report

## Abstract

**Background:**

Aortoesophageal fistula (AEF) after thoracic aortic repair is a rare and devastating complication with an extremely high mortality (33–100%). Although emergency thoracic endovascular aortic repair (TEVAR) can provide immediate hemostasis, it does not address the underlying infection and therefore is insufficient for long-term survival. A staged multidisciplinary strategy may improve outcomes in selected patients: initial hemodynamic stabilization, early source eradication (esophagectomy), and delayed reconstruction.

**Case presentation:**

A 78-year-old woman, who had undergone total arch replacement 6 years prior, presented with sudden haematemesis. Endoscopy and CT confirmed secondary AEF. Emergency TEVAR successfully stabilized bleeding. Two days later, the patient underwent thoracoscopic oesophagectomy, cervical oesophagostomy, jejunostomy, and omental flap coverage. Parvimonas micra was isolated intraoperatively, and targeted antimicrobial therapy was continued for eight weeks. After achieving infection control and nutritional optimization, presternal ileocolonic reconstruction was performed on postoperative day 69. She was discharged home 94 days after the initial intervention and remains well, free of recurrence, at 7 months post-TEVAR.

**Conclusion:**

This case highlights the critical role of a well-executed staged strategy in contributing to favorable clinical outcomes from secondary AEF. Immediate TEVAR served effectively as a life-saving bridge. Crucially, the early multidisciplinary coordination allowed for the prompt removal of the oesophageal source within 48 h, an intervention essential to prevent uncontrolled mediastinal sepsis. This sequence—haemostasis, early source control, and delayed extra-anatomical reconstruction—may provide a reasonable strategy for survival in selected high-risk patients.

## Background

Aortoesophageal fistula (AEF) is an uncommon but devastating condition associated with extremely high mortality. The underlying mechanisms of AEF formation remain poorly understood. The incidence of secondary AEF after arch replacement or thoracic endovascular aortic repair (TEVAR) has been reported to be approximately 1–2%, with mortality ranging from 33% to nearly 100% [1, 2]. Conservative treatment is uniformly fatal, and TEVAR has emerged as an effective initial measure for hemostasis [3, 4]. However, TEVAR alone does not eradicate the infectious source [3, 5, 6]. Favorable outcomes may require timely oesophageal resection, strict infection control, and eventual reconstruction through coordinated multidisciplinary care [5, 7–9]. We report a case of secondary AEF following total arch replacement that was successfully managed using a staged, team-based treatment strategy.

## Case presentation

**Fig. 1:**
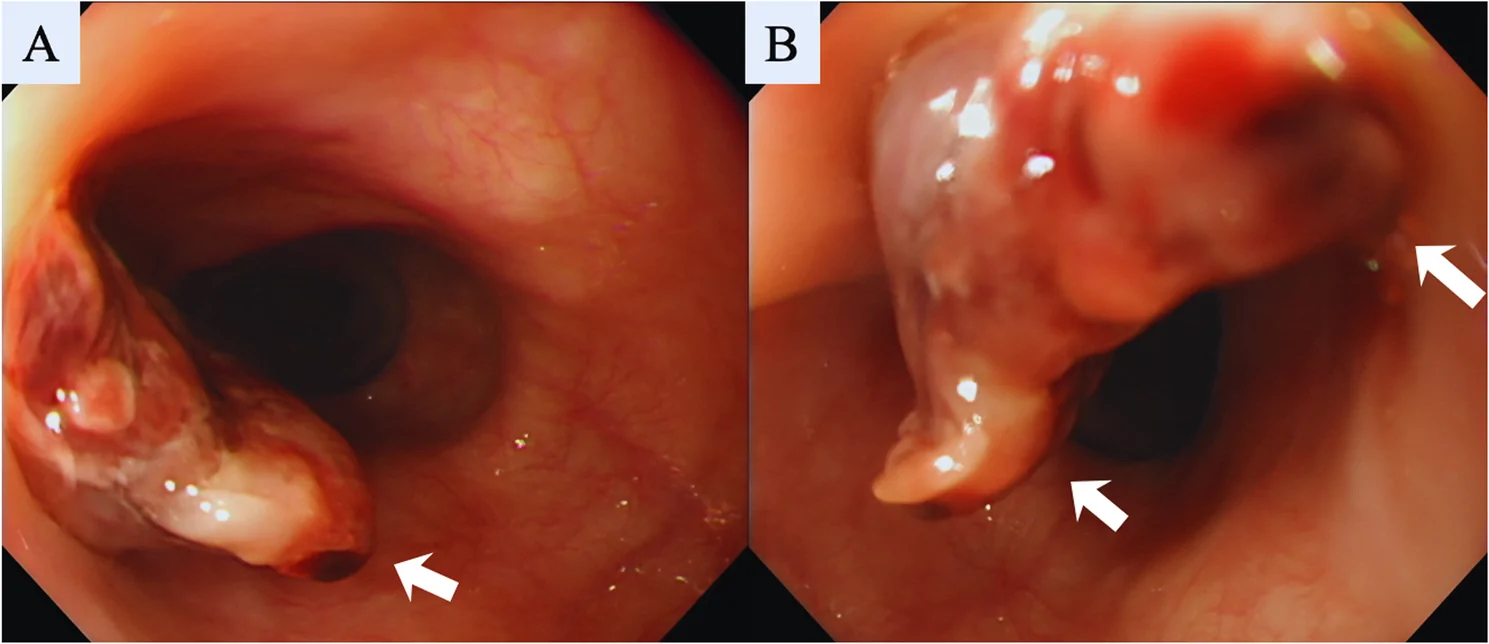
Upper gastrointestinal endoscopy findings on admission. **A** A deep ulcerative lesion in the mid-oesophagus (arrow) with surrounding mucosal destruction and adherent coagulum. **B** Closer view demonstrating necrotic debris, a protruding blood clot (arrow), and exposure of underlying tissue highly suggestive of aorto-oesophageal communication with impending active haemorrhage

A 78-year-old woman presented with sudden haematemesis. She had undergone total arch replacement using a four-branched J-graft prosthesis (22 mm; Japan Lifeline, Tokyo, Japan) for a distal aortic arch dissecting aneurysm 6 years earlier, with the distal anastomosis at proximal descending aorta, and her postoperative course had been uneventful without evidence of graft infection. She had discontinued outpatient follow-up 2 years prior to presentation. Upper gastrointestinal endoscopy at the referring hospital revealed a deep ulcerative lesion in the mid-esophagus, raising suspicion of an AEF. She was transferred to our hospital for surgical management (Fig. 1).

**Fig. 2:**
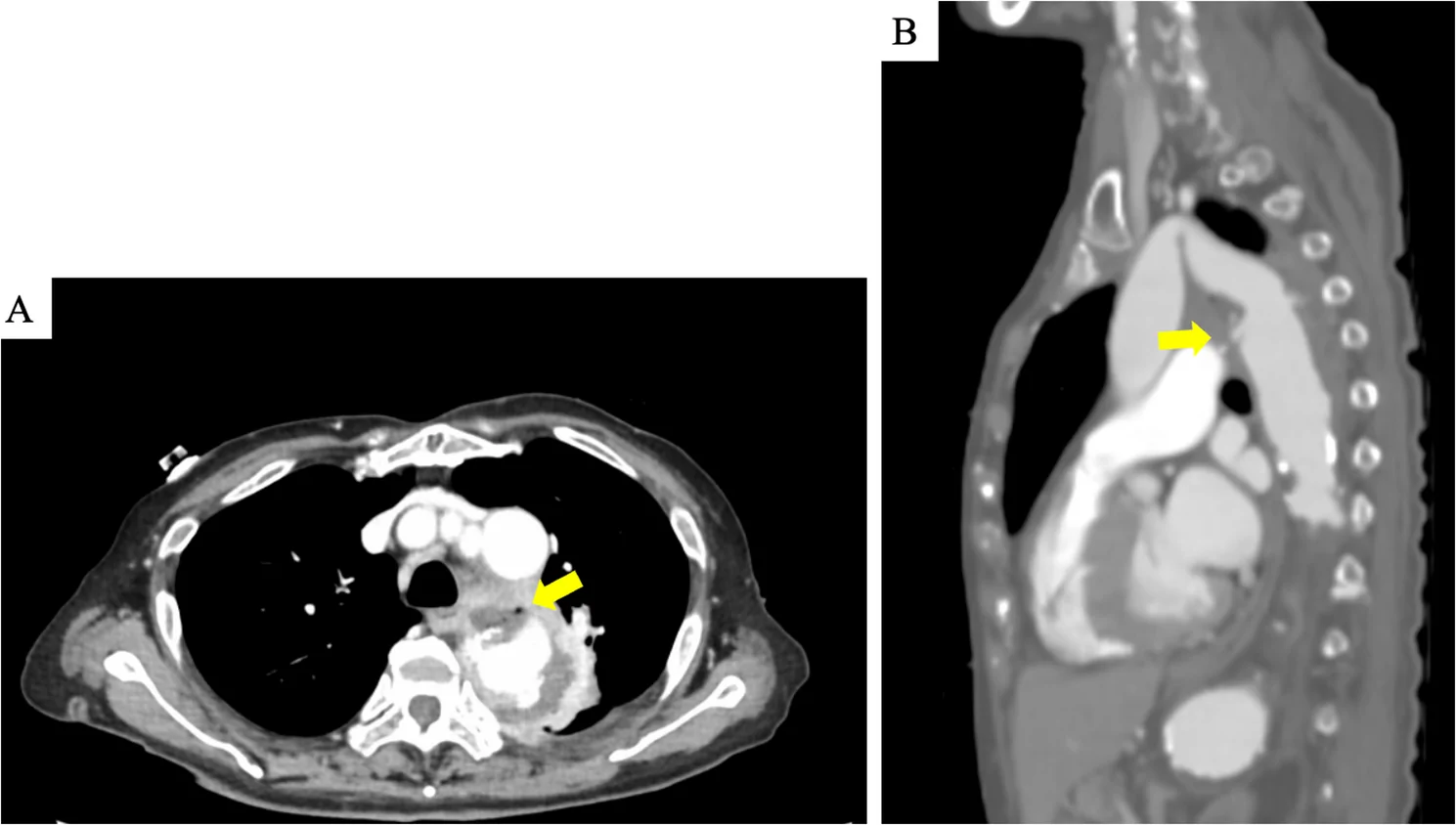
Contrast-enhanced CT findings on admission. **A** Axial contrast-enhanced CT image demonstrating peri-aortic gas and fluid collection with obliteration of the intervening fat plane between the oesophagus and prosthetic aortic graft (arrow), strongly suggestive of secondary aorto-oesophageal fistula. **B** Sagittal contrast-enhanced CT image showing the anastomotic site (arrow) adjacent to the suspected fistulous region.

On arrival at our institution, the patient was hemodynamically stable but severely malnourished (body mass index [BMI], 15.4). Laboratory investigations showed a hemoglobin level of 8.9 g/dL, a white blood cell count of 13,860/µL, a C-reactive protein level of 17.99 mg/dL, a procalcitonin level of 4.49 ng/mL, a blood urea nitrogen level of 29.4 mg/dL, and a serum creatinine level of 1.18 mg/dL. Blood cultures were negative. Contrast-enhanced CT demonstrated gas and fluid collection surrounding the descending aorta with loss of the fat plane between the aorta and the esophagus (Fig. 2). These findings strongly suggested secondary AEF. Empiric intravenous meropenem (MEPM) was initiated after blood cultures were obtained, and emergency TEVAR was performed using a Gore TAG Conformable Thoracic Endoprosthesis (W.L. Gore and Associates, Flagstaff, AZ) 34 × 34 × 200 mm thoracic stent graft, covering the suspected fistulous segment from Zone 3 on the same day. The graft was oversized by approximately 19% relative to the reference aortic diameter. Completion angiography demonstrated no endoleak (type I/III) and adequate exclusion of the suspected fistulous segment. Simultaneously, we contacted and consulted an esophageal surgery team at an affiliated institution to coordinate subsequent management.

**Fig. 3:**
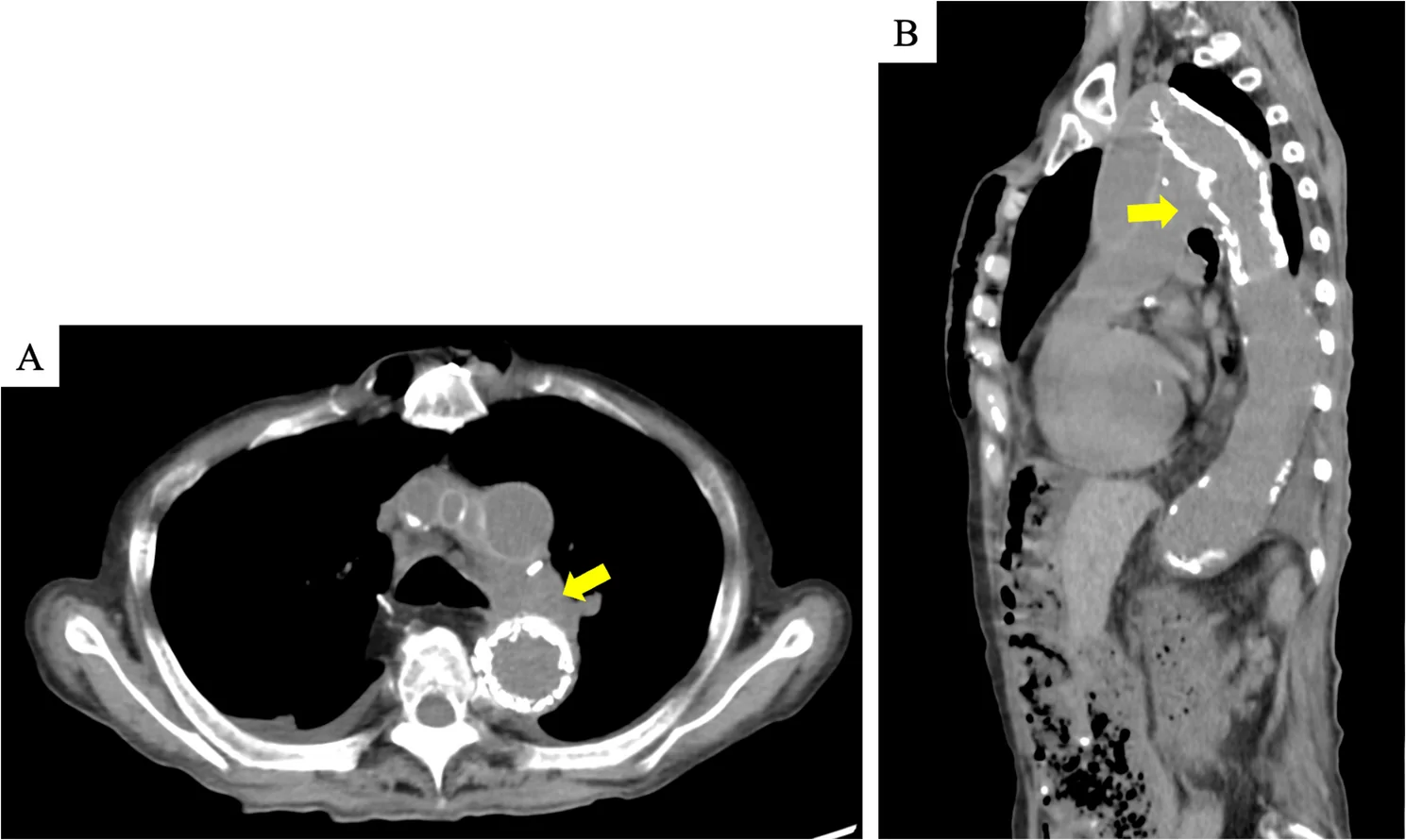
Postoperative follow-up non-contrast CT obtained after staged reconstruction. **A** Axial non-contrast CT image obtained after staged treatment demonstrating resolution of the previously observed peri-aortic gas and fluid collection seen in Figure 2, with no residual ectopic gas around the thoracic aorta, suggesting improvement of perigraft inflammatory changes. **B** Sagittal non-contrast CT image showing the thoracic aortic stent graft in situ after TEVAR and presternal oesophageal reconstruction, with no peri-graft gas or radiological evidence of persistent mediastinal infection

On postoperative day (POD) 1, the patient was weaned from mechanical ventilation. On POD 2, she was transferred to the collaborating university hospital, where thoracoscopic esophagectomy, cervical esophagostomy, jejunostomy, and omental flap coverage of the infected mediastinal area were performed. After intraoperative cultures yielded Parvimonas micra, antimicrobial therapy was de-escalated to sulbactam/ampicillin (SBT/ABPC) based on susceptibility testing and continued for a total of 8 weeks. During this period, enteral nutrition via jejunostomy and structured rehabilitation were maintained to preserve her nutritional and functional status. Her inflammatory markers gradually improved without evidence of persistent or disseminated infection. Follow-up CT demonstrated reduction of perigraft gas and fluid collection without evidence of endoleak or new perigraft complications (Fig. 3).

**Fig. 4:**
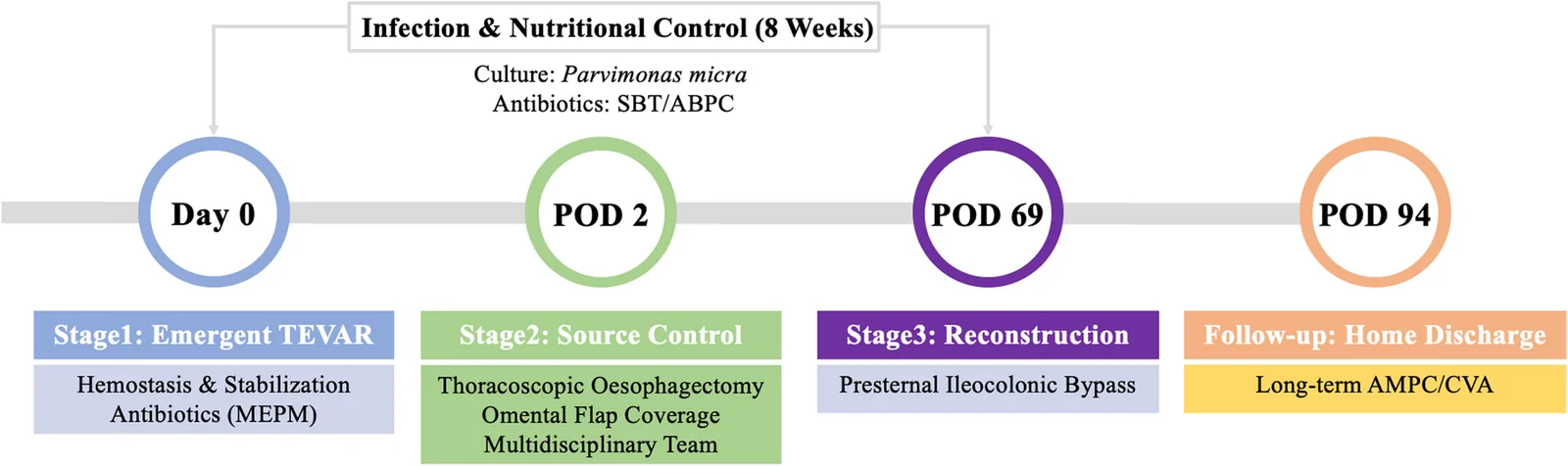
Clinical Timeline of Staged Management for Secondary Aortoesophageal Fistula. The patient underwent a three-stage management strategy after presentation. Stage 1 (Day 0) involved emergent Thoracic Endovascular Aortic Repair (TEVAR) for hemostasis. Stage 2 (POD 2) was early source control via thoracoscopic oesophagectomy and omental flap coverage performed by the multidisciplinary team. Stage 3 (POD 69) was delayed presternal ileocolonic reconstruction, which was performed after improvement of systemic and local infection (8 weeks of antibiotics) and nutritional optimization. (Abbreviations: POD, postoperative day; MEPM, meropenem; SBT/ABPC, sulbactam/ampicillin; AMPC/CVA, amoxicillin/clavulanate)

After infection control had been firmly established, presternal ileocolic reconstruction was performed on POD 69 to restore gastrointestinal continuity. The postoperative course following reconstruction was uneventful, with adequate nutritional tolerance and stable respiratory and circulatory status. After surgery, oral suppressive therapy with amoxicillin/clavulanate (AMPC/CVA) was initiated and is planned to be continued for the rest of her life. She was discharged home on POD 94 in good general condition (Fig. 4).

At the latest follow-up, seven months after TEVAR and five months after reconstructive surgery, inflammatory markers had gradually normalized, and no recurrent fever or positive blood cultures were observed during follow-up. She remained free from clinical or radiological evidence of stent-graft infection or esophageal complications.

## Discussion

AEF following thoracic aortic repair remains a rare but devastating complication associated with extremely high mortality, often reported between 33% and nearly 100% [1, 2]. Successful management requires rapid hemostasis, adequate infection control, and multidisciplinary coordination. Our case exemplifies the efficacy of this approach, highlighting that early multidisciplinary collaboration enables rapid hemostasis, effective infection control, and timely reconstruction, even in patients with severe malnutrition and acute sepsis.

### The staged strategy and its clinical rationale

Emergency TEVAR is widely recognized as the most effective initial intervention for achieving rapid hemostasis in patients presenting with massive hemorrhage. However, TEVAR alone cannot eradicate the underlying infection and is therefore associated with high rates of persistent infection, recurrent bleeding, and mortality. Long-term mortality rates of approximately 55% have been reported when TEVAR is used as sole therapy [2, 3]. The fundamental principle of definitive treatment for AEF has traditionally been complete removal of infected aorta and prosthetic material, followed by either in situ reconstruction or extra-anatomical bypass [4–6]. In the present case, although explantation of the infected descending aorta and stent graft was considered, it was deemed prohibitive due to the patient’s advanced age, severe frailty, and the high invasiveness surgical risk. Therefore, a graft-preserving staged strategy was selected as a pragmatic alternative. This approach is consistent with previously reported cases in which TEVAR was used as a bridging therapy followed by early oesophagectomy and subsequent reconstruction [5, 7, 8]. While this strategy may not represent a novel concept, the present case highlights its applicability in carefully selected high-risk patients.

### Importance of early infection source control

Early elimination of the infectious source is critical in preventing mediastinal sepsis and graft contamination. In this case, oesophagectomy was performed on POD 2 after confirming hemodynamic stability and the absence of recurrent bleeding following TEVAR. This short interval enabled early definitive infection control while minimizing perioperative risk. The timing of oesophagectomy remains controversial, as current guidelines advocate early intervention but do not define an optimal timeframe [9]. Our approach reflects a balance between urgency and patient stability, supported by multidisciplinary decision-making.

### Role of omental flap coverage and graft preservation

The omental flap provided well-vascularized biological coverage around the retained stent graft, likely contributing to local infection control and isolation of the contaminated field. Previous reports have suggested that failure of graft-preserving strategies is often associated with inadequate source control or insufficient biological coverage [5]. In contrast, the combination of early oesophagectomy and omental coverage in this case may have played a pivotal role in preventing graft infection progression. Nevertheless, graft preservation remains controversial. In general, graft explantation is recommended in cases of uncontrolled sepsis, persistent infection despite adequate source control, recurrent bleeding, endoleak-related failure, progressive graft infection, or structural graft complications such as pseudoaneurysm formation [4, 6]. In contrast, graft-preserving strategies may be considered in selected patients with controlled infection and prohibitive surgical risk, as in the present case. We acknowledge that graft preservation cannot be regarded as definitive eradication of infection, and therefore this strategy should be reserved for highly selected patients in whom radical explantation is prohibitively invasive.

## Delayed reconstruction and nutritional management

Delayed reconstruction is widely recommended to allow for sufficient infection control, resolution of systemic inflammation, and restoration of the patient’s nutritional status before definitive alimentary reconstruction. Our patient presented with marked malnutrition (BMI 15.4), underscoring the necessity of the 69-day interval of intensive enteral nutrition via jejunostomy and structured rehabilitation before reconstruction. This period allowed recovery from sepsis and nutritional optimization before reconstruction. The choice of presternal ileocolonic reconstruction on POD 69 was also strategic. This extra-anatomical route intentionally avoided the previously infected and scarred posterior mediastinum. This staged reconstructive strategy likely contributed significantly to the patient’s favorable postoperative recovery and may be an important technical consideration in similarly complex AEF cases.

### Antimicrobial strategy and long-term considerations

Because prosthetic material was retained in a potentially contaminated environment, prolonged antimicrobial therapy was considered necessary. Although long-term suppressive antibiotic therapy carries potential risks, including antimicrobial resistance and microbial substitution, it may provide a practical strategy when complete eradication of infection is not feasible. The antimicrobial strategy was determined in consultation with the infectious disease team. In this case, targeted antimicrobial therapy based on culture results was continued for 8 weeks, followed by oral suppressive therapy, with careful clinical monitoring [10].

### Follow-up strategy and limitations

Given the risk of late graft infection, lifelong surveillance is essential in patients managed with graft preservation. Our follow-up protocol includes periodic contrast-enhanced CT and monitoring of inflammatory markers. Although the 7-month outcome in this case is favorable, the relatively short follow-up period remains a limitation, and long-term durability must be evaluated [11].

### Clinical implications and conclusion

This case does not propose a novel surgical concept but rather demonstrates that a staged, graft-preserving strategy may be considered in carefully selected high-risk patients when radical surgery is not feasible. The key determinants of success include early hemostasis, prompt infection source control, effective biological coverage, nutritional optimization, carefully planned delayed reconstruction, and strict postoperative surveillance within a multidisciplinary framework.

## Data Availability

The datasets used and analysed during the current study are available from the corresponding author on reasonable request.

## Abbreviations

AEF: aortoesophageal fistula
TEVAR: thoracic endovascular aortic repair
BMI: body mass index
MEPM: meropenem
POD: postoperative day
SBT/ABPC: sulbactam/ampicillin
AMPC/CVA: amoxicillin/clavulanate
